# Fundamentals of Transport in Polymers and Membranes—Honorary Issue for Professor Giulio C. Sarti

**DOI:** 10.3390/membranes16030108

**Published:** 2026-03-18

**Authors:** Maria Grazia De Angelis, Matteo Minelli

**Affiliations:** 1 Department of Civil, Chemical, Environmental and Materials Engineering (DICAM), Alma Mater Studiourm—University of Bologna, 40131 Bologna, Italy; grazia.deangelis@unibo.it; 2 Institute for Materials and Processes, School of Engineering, University of Edinburgh, Sanderson Building, Robert Stevenson Road, Edinburgh EH9 3FB, UK

This Special Issue of *Membranes* celebrates the scientific achievements, international vision, and collaborative spirit of Professor Giulio C. Sarti, whose career has had a profound and lasting impact on the membrane science community. Over the years, his research has spanned a broad range of topics, from fundamental studies on anomalous diffusion phenomena in polymers [1,2] to applied investigations in membrane-based gas separation [3,4], water treatment [5,6], and bioseparations [7,8].

A defining feature of his career has been the strong network of international collaborations he has established and cultivated across continents, fostering scientific exchange and building enduring partnerships between research groups worldwide. By bridging theory and application, Professor Sarti has significantly advanced both our fundamental understanding of transport phenomena and the practical development of separation technologies.

Perhaps the most distinctive legacy of Professor Sarti’s scientific work has been his remarkable ability to tackle complex, scarcely understood problems with an approach combining conceptual clarity and rigorous analysis. His unique ability to make challenging phenomena accessible without sacrificing scientific depth has inspired generations of researchers over more than 40 years. To acknowledge this aspect, this Special Issue is devoted to “Fundamentals of Transport in Polymers and Membranes”, a theme that closely reflects the core of Professor Sarti’s legacy.

The Special Issue brings together contributions across several interconnected themes, successfully addressing key questions and topics related to membrane processes, with research contributions from both experimental and modeling perspectives. We present 15 significant papers by renowned experts in this field from a range of countries in Europe and from the US.

Some of the contributions cover the fundamentals of mass transport in polymers and polymer-based materials, with particular attention given to diffusion mechanisms and sorption phenomena. The main research aim across the different papers is not only to characterize the properties of the materials of interest, either in polymer commodities, new formulations, or newly developed composites, but also to provide useful and more general indications in a broader context, using dedicated modeling approaches or deriving structure–performance correlations.

The article by Borrmann et al. (contribution 1), “Water Sorption in Glassy Polyvinylpyrrolidone-Based Polymers”, is focused on the analysis of gas sorption in two polyvinylpyrrolidone-based materials often employed in the food and pharma industries. The resulting water solubility and sorption kinetics, showing an activity range spanning from 0 to 90%, are reported and analyzed thoroughly. The large water uptakes observed in the polymers can induce phase change, from a glassy to a rubbery state. Dedicated modeling efforts are then employed to model the solubility in the equilibrium rubbery phase using the PC-SAFT equation of state and in the glassy non-equilibrium phase by taking advantage of the Non-Equilibrium Thermodynamics for Glassy Polymers (NET-GP) approach originally proposed by Doghieri and Sarti [9]. The sorption kinetics is also modeled, considering a free volume approach embedded in the Maxwell–Stefan framework for mass transport. The approach presented describes accurately the experimental data, revealing the solidity of the model and its ability to represent a wide range of conditions.

Hallinan et al. (contribution 2) investigate water solubility and diffusivity behavior in different grades of a conventional polymer, polystyrene, well known for its hydrophobicity, in “Effect of Polystyrene Synthesis Method on Water Sorption and Glass Transition”. Interestingly, the authors reveal how the synthesis method may notably affect water sorption and transport, as polymer molecular weight and chain ends play a significant role. Solubility varies by a factor 3 to 4, while diffusivity changes even by an order of magnitude. Although the solubility of water remains low, the presence of dissolved water molecules can alter its properties, and an appreciable glass transition temperature decrease is observed with increasing water activities, as determined using calorimetric or dynamo-mechanical experiments.

The work by Baldanza et al. (contribution 3), “Chemical Vapour Deposition Graphene–PMMA Nanolaminates for Flexible Gas Barrier”, is focused on the development of a new concept for nanocomposite materials for gas barrier applications, taking advantage of 2D materials and graphene in particular. The layer assembly is carried out using an iterative lift-off/float-on process combined with wet depositions, fabricating thin membranes of PMMA and graphene arranged in an extremely ordered fashion. That allows us to exploit the graphene barrier potential at its best, even with extremely low loadings of the 2D material. The measured permeabilities of CO_2_ and O_2_ are approximately 20-fold lower than the pristine PMMA, indicating how these laminates outperform traditional nanocomposite polymer/2D materials in terms of gas barrier properties.

Membranes for gas separation also represent one of the key applications considered by Professor Sarti during his career. This is a topic covered in several different contributions in this Special Issue. Papers highlight advances in material design and process simulation and optimization, as well as sustainability considerations, focusing on a range of applications, mainly related to post-combustion carbon capture (i.e., CO_2_/N_2_ separation), natural gas sweetening (CO_2_/CH_4_ or H_2_S/CH_4_), and air separation (O_2_/N_2_).

Signorini et al. (contribution 4), in “Permeation of Ternary Mixture Containing H_2_S, CO_2_ and CH_4_ in Aquivion^®^ Perfluorosulfonic Acid (PFSA) Ionomer Membranes”, discuss the use of a proton exchange membrane for gas separation purposes, specifcally for the removal of acid gases (CO_2_ and H_2_S) from natural gas. The paper addresses in detail the effect of the water humidity, temperature, and pressure of the feed stream, revealing that the membrane material, Aquivion, when exposed to humid feeds, is characterized by a remarkable ability to discriminate penetrants based on their water solubility.

Longo et al. (contribution 5) report the development and characterization of a novel membrane material obtained by blending Matrimid^®^ polyimide, a classical benchmark for gas separation, with a polymer of intrinsic microporosity in the form of a thin-film composite. In “Thin-Film Composite Membranes Based on the Polymer of Intrinsic Microporosity PIM-EA(Me_2_)-TB Blended with Matrimid^®^5218”, the authors share in detail the effect of the porous substrates on the resulting permeability and selectivity performance of the resulting membranes and identify ageing issues, most likely enhanced by the reduced thickness of the active layers.

For the contribution “Mixed-Matrix Membranes Loaded with a Porous Organic Polymer With Bipyridine Moieties”, Rico-Martínez et al. (contribution 6) developed a mixed-matrix membrane derived from three aromatic polyimides, combined with porous organic polymer, a highly microporous moiety. The resulting membranes were stable and showed good mechanical properties as a consequence of the excellent compatibility of the two phases. Permeability was shown to be significantly enhanced through the presence of the porous filler, ensuring good selectivities for different gas separations.

Han and Ho (contribution 7) provide a different prospective in “Moving beyond 90% Carbon Capture by Highly Selective Membrane Processes”, where a techno-economic analysis of a CO_2_/N_2_ separation process is reported for post-combustion carbon capture. Based on the use of a highly CO_2_-selective facilitated transport membrane, different possible process schemes are inspected, achieving optimal values for CO_2_ capture (as high as 99%) at low costs while still meeting the purity criteria required.

To acknowledge the essential contribution given by Prof. Sarti in developing models for the fundamental description of polymers and membranes [10,11,12], the Issue explores also modeling approaches across multiple scales, from molecular-level descriptions to continuum and process-scale analyses, providing insights into gas transport behavior in polymers and related properties.

In the paper “Effect of Packing Nonuniformity at the Fiber Bundle–Case Interface on Performance of Hollow Fiber Membrane Gas Separation Modules” (contribution 8), Sun et al. address the practical challenge of improving the design of membrane modules, particularly for applications such as gas separation and carbon capture. To this end, they develop computationally efficient simulation models of flow distribution based on a planar approximation, which closely reproduces the results of full 3D simulations while significantly reducing the computational cost. The simulations reveal the detrimental effects of poor fiber packing at the bundle–case interface, which can substantially affect the overall performance of the module.

A similar approach is followed by Murmura et al. in the paper entitled “Toward Minimal Complexity Models of Membrane Reactors for Hydrogen Production” (contribution 9), which simplifies the 2D model for methane steam-reforming membrane reactors, enabling the determination of membrane reactor performance at an extremely low computational cost and with a good degree of accuracy. A 1D approach is thus developed, considering an enhanced Sherwood number, depending on the stoichiometry of the reaction considered, relying on the fact that the same expression for Sh can be used regardless of the reactive system. The resulting simulation tool is thus extremely versatile.

Professor Sarti made an indelible contribution to the development of models describing gas solubility and transport in solid materials, including the Non-Equilibrium Lattice Fluid (NELF) model [9,13] and the Standard Transport Model for diffusion [14]. Following in his footsteps in the field of glassy polymers, Brandani introduced the RALF model for rigid sorbents, extending the Lattice Fluid theory to rigid porous structures, in the contribution entitled “The Rigid Adsorbent Lattice Fluid Model: Thermodynamic Consistency and Relationship to the Real Adsorbed Solution Theory”, enabling its connection to the classical Real Adsorbed Solution Theory (RAST) (contribution 10). The approach provides a suitable modeling tool for the prediction of multicomponent adsorption based on pure component data as an alternative to conventional methods.

Marshall et al. were also inspired by the NELF framework in proposing the Dry Glass Reference Perturbation Theory, a self-consistent description tailored to glassy polymers, applied to the separations of complex liquid mixtures in the paper “Dry Glass Reference Perturbation Theory Predictions of the Temperature- and Pressure-Dependent Separations of Complex Liquid Mixtures Using SBAD-1 Glassy Polymer Membranes” (contribution 11). Interestingly, the model developed proved to be effective in the prediction of mass transport of mixtures of organic molecules over a wide range of temperatures and pressures.

A different modeling approach is the one proposed by Neyertz et al., who simulated the transport of gases following a molecular-level method in their study “Molecular Characterization of Membrane Gas Separation under Very High Temperatures and Pressure: Single- and Mixed-Gas CO_2_/CH_4_ and CO_2_/N_2_ Permselectivities in Hybrid Networks” (contribution 12). Molecular dynamics simulations were indeed employed to predict mixed-gas permeability in glassy polymers and hybrid structures, identifying the relative contribution of solubility and diffusivity in a wide range of conditions.

Multiscale strategies that bridge molecular simulations and continuum theories for solubility, diffusivity, and permeability, including the NET-GP, are comprehensively analyzed and discussed by Ricci et al. in “Modelling Sorption and Transport of Gases in Polymeric Membranes across Different Scales: A Review” (contribution 13). The paper highlights the ongoing integration of modeling tools across different length scales for sorption, diffusion, and permeation in polymers.

Last but not least, this Special Issue features interesting contributions on bioseparations and membrane-based processes, illustrating the expanding role of membrane science in biotechnology and environmentally relevant applications, a domain also widely covered by Professor Sarti.

Lalli et al. present the paper “Use of the Dispersion Coefficient as the Sole Structural Parameter to Model Membrane Chromatography” (contribution 14), in which they demonstrate how breakthrough curves in membrane chromatography can be accurately predicted using the experimental axial dispersion coefficient, rather than assumed pore size distributions, which provides the only physically grounded description of dispersion phenomena in porous membranes.

Chemical engineering can go beyond that, tackling the biomanufacturing industry, where the purification of viral vectors for gene therapy applications is an ongoing challenge. Fan et al., in their article “Purification of Adeno-Associated Virus (AAV) Serotype 2 from Spodoptera frugiperda (Sf9) Lysate by Chromatographic Nonwoven Membranes”, demonstrate for the first time nonwoven ion-exchange membrane adsorbers for the capture and purification of AAV2 from crude Sf9 lysate (contribution 15).

In conclusion, the outstanding contributions in this Special Issue provide solid and relevant answers to a wide variety of interesting scientific questions, covering different fields of membrane science.

The Guest Editors hope that this Special Issue will provide useful information and context to young and established scientists in the field to inform future work and serve as a source of inspiration, as Professor Sarti has been and remains for a number of former students, colleagues, and international collaborators through his extraordinary scientific journey.
